# Miscellanies

**Published:** 1831-04-01

**Authors:** 


### MISCELLANIES.

### XIX.

### Cases of Foreign Bodies stuck in the Œsophagus.*

*Case* 1. In November, 1826, a man, after eating and drinking to excess at a village feast, was attacked with vomiting, and a sense of great uneasiness in the line of the œsophagus; he could neither swallow his saliva, nor any other liquid; respiration became difficult, and he was perfectly unable to articulate. A gentleman who was summoned, thinking him merely drunk, prescribed some simple diluent drink, and walked off. But suffocation seemed impending, and M. Boileau, of Nancy, was summoned. The patient indicated by signs that something gave him great pain in the region of the throat. On introducing an elastic gum bougie into the pharynx and œsophagus, it was

* Archives Générales, Août, 1830.

stopped in the superior part of the latter. M. Boileau then took a bougie 14 or 15 inches in length, and fixing to one of its extremities some linen smeared with lard, he passed it into the œsophagus, and by its means pushed down the obstructing body towards the stomach. The patient immediately cried he was saved, and suffered no more inconvenience worth mentioning.

*Case* 2. On the 2d Sept. 1829, a woman applied to M. Boileau on account of pain in the middle of the neck. She said that on the 31st August she had swallowed a mutton bone, since which time she had had the pain, and been unable to swallow liquids or solids. By means of the elastic gum bougie, M. Boileau became satisfied of the presence of the foreign body in the œsophagus, on its right side and upper part. He then took an instrument *(tige en haleine)* and tying to one end a conical piece of sponge, about the size of a pullet's egg, he bound it tight with strong twine, which he allowed to remain on it for a quarter of an hour. He then unrolled the twine, smeared the sponge with lard, and passed it down the œsophagus into the stomach. Having left it there for a few minutes, he withdrew it gently, and as the broad end of the sponge travelled up the œsophagus, it caught the foreign body and drew it up into the mouth. In seven or eight days the pain in deglutition was entirely gone. The piece of bone thus removed, was part of the scapula of a sheep; it was fifteen lines long and six broad.

*Case* 3. A man laid a wager that he would swallow a five franc piece, intending at the time to conceal it in his mouth. In the counterfeited efforts at deglutition, however, the trick failed, and the franc piece did really pass into the pharynx, where it stuck. The man now applied to M. Dupuytren. He attempted at first to extract it with a pair of curved forceps, but failed. He then took a very simple instrument, composed of a *tige en haleine*, twelve to fifteen inches long, and of sufficient diameter to be firm. At one end was a little rod *(virole)* stretching beyond the instrument, in the form of a small cylinder. Two metallic rings united at a portion of this circumference and separated about half an inch at their opposite side, were fixed by their concave surface and point of junction to the little suebal rod. This instrument being introduced into the pharynx and œsophagus, was passed to the level of the foreign body, the ring carried below it, and when the instrument was withdrawn, the five-franc piece was entangled in and extracted with it.

In his clinique on the above case M. Dupuytren mentioned two others which had been communicated to him by Baron Larrey. A soldier having made a wager that he would swallow a five-franc piece did actually do so. The patient's health suffered little or not at all, but it was not till twenty years afterwards that the coin passed by stool; it was then blackish and much diminished in size. Another soldier having made a similar wager, the five-franc piece passed slowly down the pharynx and œsophagus, and stuck about its middle. A number of attempts at extraction remained, but none succeeded. Deglutition was not impossible, and the œsophagus was left undisturbed for six months, when the man was attacked with low fever, of which he died. On *dissection* the five-franc piece was found at the inferior part of the œsophagus, above the cardiac orifice of the stomach. The walls of the œsophagus here were thickened, indurated, and in a state of suppuration.

---

### XX.

### SCHLOSS'S ANATOMICAL PLATES.*

WHATEVER may be the faults of JOHN

---

* 1. Anatomical Atlas of Dr. M. J. Weber, Professor of the Royal Prussian University, Frederic William, at Bonn. Plates I. to XVI.

BULL, he can hardly be accused of blindness to foreign merit, or of pertinacious obstinacy in refusing to encourage that which is not of exclusively British growth. England is the mart for the produce of the world, whether it spring from the head or the hands; and deserving foreigners can rarely complain of not having experienced liberal encouragement in this land of wealth, liberty, and industry. We were led to this reflection by a glance at the work before us. The cultivation of practical anatomy in this country has of late been involved in such difficulties, not to say dangers, that it is not surprising if our knowledge on the subject is inferior to that which obtains elsewhere. The French, availing themselves of the opportunities which they possess, have given to the study of anatomy amongst them a degree of precision and accuracy, which, in many points, it does not boast of with us. The Germans, again, excel the French, and the anatomical researches of Soemerring, Meckel, Tiedeman, and others, have conferred a very high character on the anatomical science of the country.

Where bodies are procured with difficulty, risk, and expense, the student must necessarily rely more or less upon plates. Mr. Lizars deserves much credit for the attempt which he has made to meet this want on the part of the professional student; but although there is great merit both in the design and in the execution of his Anatomical Atlas, it cannot be concealed that it has many faults. Where constant reference can be made to the dissected *subject*, delineations of the parts must necessarily be more correct, than where such reference is not very readily obtained. It is for this reason, no doubt, that the anatomical plates of the German authors are so much more accurate than our own, and it is for this reason that we strongly recommend the Atlas whose title heads this article to the attention of the profession. We have looked over the plates with critical eyes, and we candidly avow that we have little fault to find with them—they are reasonable in price, the execution is admirable, and the information which they convey to be depended on. We recommend them alike to the young student, and the more experienced surgeon; we have hung them up in our own library, and we advise our brethren to purchase them and do the same. A master cannot make a more valuable present to his apprentice, a father to his son, than by giving him these plates to study, before or after he begins to read the still more accurate book of Nature. We hope that Mr. Schloss will follow up his design, and give a connected series of plates, embracing all the points of human anatomy, which it is right and profitable to know, and we dare to promise that if he do so, he will find his reward in the merited patronage of an English public. We shall mention the Parts from time to time as they are published, in order that our country readers may know the extent, character, and progress of this very useful work.

2. Explanation of the Anatomical Atlas. London: published by A. Schloss, Foreign Bookseller, 44, Southampton Buildings, Chancery Lane, 1831.

---

### XXI.

### CASE OF OSSIFICATION OF THE MITRAL AND AORTIC VALVES. By PATRICK CLINTON, M.D. &c.*

ELLEN PRINTER, æt. 25, of delicate constitution, who had often miscarried, was visited Sept. 19th, 1828. She had dyspnœa, frequent violent palpitations, orthopnœa, a hard cough, with sometimes bloody expectoration, and œdema of the feet. The contractions of the ventricles were accompanied with a loud and distinct bruit de soufflet, heard all over the anterior part of the chest, on account of the emaciation. She took gentle laxatives, with tincture of digitalis. On the next day the palpitation was very violent, and its impulse observed to be strongest between the

* Dublin Transactions, Vol. I. Part I.

third and fourth cartilages of the left side. Percussion gave a dull sound in the region of the heart. The right arm was affected with severe pain from the shoulder to the tips of the fingers, and she stated that, four months previously, she had been affected with paralysis of the right side. On the 24th, she was easier. The heart's action was very irregular, the ventricular stroke very long, that of the auricles hardly distinguished, and followed by an intermission equal in duration to both. On the 25th, the pulse was 100, the beat of the heart very irregular. First there were two or three strokes with a long intermission between them, and then an equal or a greater number in rapid succession. The expectoration was less bloody, the respiration puerile under the left clavicle; the edge of the liver was felt below the navel. By the 2d Oct. the legs were swelled up to the knees; the impulse of the heart was less, the noise loud and peculiar, that of the ventricles resembling the rolling of a cart, and the pronunciation of the word *thürla* conveying some idea of it. There was sibilous rattle in the chest. On the 8th, she was much worse, on the 10th and 11th, the respiration was heard only at the upper part of the chest, the dyspnœa increased, and at 9, p.m. she died.

*Sectio Cadaveris.* The liver was much enlarged and very hard, its external surface appearing as if minute grains of sand were closely imbedded in it. The right lung adhered to the pleura costalis; there was no water in either pleural cavity. In the pericardium was upwards of a quart of greenish-yellow transparent fluid, with a very few flakes of soft lymph floating through it. The heart, especially the curricular portion, was distended by a quantity of clotted blood. The mitral valves were completely ossified, projecting towards each other, so as actually to meet in part of their length, and form in the remainder a mere chink, not large enough to admit the handle of a scalpel. The edges of the valves were very irregular, nodulated, and thickened; there was much ossification under the lining membrane of the parts in their vicinity; and the columnæ carneæ and chordæ tendineæ were thicker and stronger than natural. The auricle was thickened in its parietes, and much dilated in its cavity. The ventricle was rather thinner than natural. The aortic valves were imperfectly ossified, but considerably thickened, and standing up so as nearly to close the passage into the vessel. There were two or three minute points of ossification in the aorta, about an inch from the heart. The tricuspid valves were thickened and imperfectly ossified, and stood up a little from the sides of the ventricle. The right ventricle and the valves of the pulmonary artery were healthy; the right auricle was thickened and enlarged, but not so much as the left. The lungs were not cut into, but externally they appeared sound.

This case is of interest in connexion with other instances of contraction of the left auriculo-ventricular opening with which we have presented our readers on former occasions. Our author makes a number of remarks on this and some other cases, but the following are all that we need notice.

"With respect to treatment, that which I have found most beneficial was the administration under different forms, of digitalis purpurea. In the advanced stages of the disease, in which alone I have had an opportunity of employing it, bloodletting afforded only temporary relief, and in some instances, it seemed to hurry on the fatal termination. However, I have no doubt, that when employed in due season, it may, in conjunction with proper regimen, prove a most valuable remedy. It may either retard the progress of the primary disease, or prevent its most fatal consequences. Thus it may prevent apoplexy in cases of hypertrophy of the left ventricle; and in all cases it may remove inflammation of the serous membranes, and thereby prevent effusion into their cavities. With a view to prevent the latter accident, it is obvious, that exposure to cold ought to be carefully avoided by those who are affected with disease of the heart.

The following case is an instance of the utility of digitalis, combined with calomel, in treating the advanced stages of the disease.

Anne Moor, had cough accompanied by a copious expectoration of mucus tinged with blood, orthopnœa, violent palpitation, pulse 160 and irregular, œdema of the lower extremities, fulness of the right hypochondrium, and no sleep. She took a pill composed of calomel and digitalis three times a day, until it produced ptyalism. In a short time the expectoration ceased, the swelling of the legs was removed, the palpitation diminished, the pulse, which was still irregular, was reduced to 100; she was able to lie down in the horizontal position, and sometimes enjoyed a sleep of five or six hours duration. In the course of this woman's illness, the same treatment was several times repeated, and with similar good effects.

On examination after death, there were found ossification of the mitral and aortic valves, adhesion of both lungs to the pleura costalis, and dropsy of both sides of the chest and of the pericardium. This woman had been bled for inflammation of the lungs, about three months before she became my patient."

---

### XXII.

### CONGENITAL INCONTINENCE OF URINE.

Dr. OTTO, of Pennsylvania, has published some cases of this kind in our NORTH AMERICAN cotemporary of October last, which we shall here notice.

*Case* 1.—This was a boy, ten years of age, who had been afflicted with incontinence of urine from his birth, and for which no remedies had been tried. The Doctor ordered a strong infusion of uva ursi to be made (an ounce to the pint of water) and a wine-glassful to be taken four times a-day. He was also directed to take 15 drops of the muriated tincture of iron thrice daily. The boy improved rapidly under this treatment, and got entirely free from his loathsome ailment.

*Case* 2.—This was a lad, 12 years of age, who was in the habit of wetting his bed about once a week. He had frequent desire to make water; and discharged but little at a time. If he finds not a convenient place, he sometimes is forced to pass water, even in the day, among his clothes. The same remedies as are above detailed were ordered. His complaint fluctuated, but, upon the whole, he improved. A large blister was applied to the sacrum, with some additional benefit. The tartar emetic ointment was next applied. Still a cure was not effected. The upland sumach (rhus glabrum) was substituted for the uva ursi, and oil of turpentine was given twice a-day. By these means he so far recovered, that he was discharged from a charitable institution, and was bound apprentice to a trade.

*Case* 3.—This was a young female, aged 9 years, residing in the same institution with the preceding patient for three years, and has all that time been liable to incontinence of urine, which has probably continued since birth. She seldom misses wetting her bed a single night. She makes water frequently in the day, but not involuntarily, except when frightened, when she is quite unable to retain it. She was directed to take a wine-glassful of the decoction of the uva ursi, and five drops of the muriated tincture of iron as often. No improvement taking place, the drops were increased to ten, after which she mended rapidly, and did not wet her bed for several months.

Two or three other cases, nearly similar, are detailed by our author; but the above are sufficient.

---

### XXIII.

### LITHOTRITY.

Mr. COSTELLO read a paper at the Westminster Medical Society, a few evenings ago, on the subject of lithotrity, and produced a very excellent example of the good effects of this operation—namely, a patient turned of 60 years of age, who had been cut in the usual way, some years ago, by Mr. Attenburrow, of Nottingham, but who afterwards became the subject of calculus, and was reduced to the lowest ebb of misery and suffering by the tortures which the stone occasioned. When this man (who was a respectable farmer) arrived in London, he was so broken down in health, that Mr. Costello was afraid to operate; but by tranquillizing the bladder by means of opiate injections, &c. the patient was brought into a more favorable condition, and in three sittings a large stone was perforated, crushed, and discharged. The patient appeared at the Society in perfect health, though the last operation had been performed only a few days previously. He did the operator great credit. Mr. Costello exhibited the instrument used on this occasion, and answered very candidly a number of questions asked by different members of the Society. Mr. C. being a countryman of our own, ought to be encouraged by the profession of these realms.

---

### XXIV.

### NAVAL SURGEONS.

*To the Members of the Royal College of Surgeons in London.*

GENTLEMEN,—In obedience to your resolution of the 8th inst. we have this day waited on his Grace the Duke of Devonshire on the subject of the exclusion of the surgeons and assistant-surgeons of the navy from his Majesty's levees. His Grace said he had great pleasure in being able to authorise us to communicate to you the following answer: — "That his Majesty entertained the kindest feelings towards the surgeons and assistant-surgeons of his navy, that the order complained of was rescinded, and that in future those officers would be admitted to the levees through the Lords of the Admiralty." Offering you our warmest congratulations on this result, we have the honour to remain, your obedient servants,

GEORGE WALKER.
THOMAS KING.

209, Piccadilly, March 17, 1831.

### XXV.

### IMPORTANT TO SURGEONS.

Home Circuit, Maidstone, March 18.
(Before Mr. Baron Bayley and a Special Jury.)

THE APOTHECARIES' COMPANY v. RYAN.

This was an action brought by the Master and Wardens of the Apothecaries' Company to recover a certain penalty, fixed by Act of Parliament, from the defendant, alleged to have been incurred by him for practising as an apothecary without a license from the Apothecaries' Company. This case is exceedingly important, as there is no instance on record in which the Apothecaries' Company have tried this question with a Member of the College of Surgeons.

Mr. Gurney, with whom was Mr. Thessager, in stating the plaintiff's case, observed that by the 55 Geo. III, it was enacted that any person practising as an apothecary, not having a license from the Apothecaries' Company, was liable to a penalty of 20*l*. The defendant is a surgeon practising at Farningham, and has been in the habit of attending patients, and compounding medicines, not holding a license from the Company, although a Member of the College of Surgeons. Several witnesses whom Mr. Ryan had subpœnaed were called, and considerable laughter was created in the Court by the different questions which were put to distinguish between what were medical and what surgical cases.

Mr. Platt, with whom was Mr. Clarkson, in a very luminous speech, submitted to the Jury that the defendant, as a Member of the Royal College of Surgeons, possessed the right, both by law and usage, to furnish medicine in both surgical and medical cases. All the cases, however, in which the defendant was engaged, were *de facto*, surgical cases, although he admitted it might have been necessary to have given medicines; yet ultimately the patient must have had recourse to his services as a surgeon. The defendant had never sent in any bill as an apothecary, and had never made up any physician's prescriptions. It was quite absurd so suppose that a surgeon could exercise his calling without prescribing medicines, or rather, without preparing the patient for the services of the surgeon. The learned counsel, in conclusion, contended that the Statute of the 32d of Hen. VIII. which empowered physicians to vend medicines, also included surgeons, surgery being a special member and part of physic.

Mr. Baron Bayley left it to the Jury to say whether the cases proved were cases of pure surgery or not. The distinctions between the different modes of practising was originally this:—The physicians advised, the apothecary mixed up the medicine, and the surgeon performed the operations. It seemed to him that the Act which had been referred to contemplated the advice and making up medicine to be the practice of an apothecary. There was a great distinction between a surgical case and one in which a surgical operation might become necessary.

Mr. Baron Bayley—in answer to a question by the Jury.—I think the words of the Charter confine the practice to external diseases.

The Jury then said that, under the direction of his lordship as to the Charter, they found it their duty to find for the plaintiffs, because there was no external injury in the first instance.—Verdict for the plaintiffs.

The learned Judge summed up decidedly in favour of the Apothecaries' Company, and laid down the law in such a manner, that the Jury were compelled, as it were, to return a verdict for the defendant. The Judge declared that in his opinion two of the cases, water in the chest, and inflammation of the liver, were decidedly medical, and that therefore the defendant had practised as an apothecary. The Judge further said that, in his opinion, the law laid it down that no surgeon could legally make up and send out medicines, excepting such cases as were mentioned in the Charter of Charles II, which exclusively relate to external diseases.

---

### XXVI.

### Mr. Hawkins on the Guaco.

The following letter, which appeared in the Medical Gazette, is deserving of attention. As it is short, our readers will excuse us for inserting it entire from our contemporary.

" *To the Editor of the London Medical Gazette.*

Sir,

In the Medical Gazette, vol. vi. p. 507, there is a short notice of a paper which was read at the College of Physicians, containing an account of some experiments, which I had been enabled to make through the kindness of the president with the 'mikania guaco,' a plant which is said by the inhabitants of South America, and some of the West India islands, to prevent or cure the bites of poisonous serpents, and rabid dogs. The quantity which I possessed was not sufficient to investigate the subject completely, but I ascertained that it did not *prevent* poisonous serpents from biting animals which were under the influence of the reputed preservative, nor did it seem to do more than mitigate the symptoms of rabies or hydrophobia; it did not seem to retard the fatal result either in man or the dog. Still, however, the belief in the efficacy of the medicine is so strong—it is so widely diffused—and has continued so long, that although I am inclined to think the accounts we have received are much exaggerated, it is well deserving of further trial, as even palliation of the frightful symptoms of hydrophobia is yet a desideratum. On this account I avail myself of your journal to inform the members of our profession that I am in possession of some more of the plant, which has been sent to me by Sir Robert Kerr Porter, from the Caraccas, some of which I shall be happy to give to any medical man in London, or its neighbourhood, who meets with a case of hydrophobia, in order that its virtues may be again put to the test.

It might appear superfluous, perhaps, to allude to the necessity of accurately distinguishing hydrophobia from other diseases; and yet it is singular to what an extent the apprehensions of the public have been erroneously led with regard to the prevalence of this disease. During the last year, rabies was particularly prevalent among dogs, and the newspapers were constantly full of accounts of mad dogs and cases of hydrophobia, and yet when a return was ordered by the House of Commons of the number of cases which had occurred in the preceding year in all the metropolitan hospitals, it appeared that the instance which I alluded to in my paper, in which the guaco had been tried, was the only one which had been admitted into them. One case, which had been detailed most circumstantially and frequently by the daily press, was that of a poor man, who had died, as I learned, from the physician who attended him, of delirium tremens from drunkenness; nor were these mistakes made only by the public, but unfortunately some medical men have themselves contributed to encourage the popular fears by their ignorance of the disease: one gentleman, for instance, in giving his evidence at a Coroner's Inquest, expressed his opinion that the patient had died of hydrophobia, and the verdict was recorded upon this evidence, the patient having in reality died a *few days* after the receipt of the injury, no doubt (if the report of the evidence was correct) from the irritation of a lacerated wound.

It is not, therefore, so much the frequency of hydrophobia as the extraordinary, and uniformly fatal nature of the complaint, which induces me to request you to give effect to Sir R. K. Porter's humane exertions, by giving publicity to this letter, in order that the medicine may be employed whenever an opportunity occurs.

I am, Sir,

Your obedient servant,

CÆSAR HAWKINS."

---

### XXVII.

### LONDON COLLEGE OF MEDICINE.

THE following is the basis on which the new college is proposed to rest; or rather the code of principles by which it is to be governed. The paper only reached us just as this last sheet of the Journal was going to press, and therefore we have no time nor room for comments.

"All medical gentlemen now legally qualified to practise in either branch of the profession shall be deemed eligible candidates for the Diplomas of the LONDON COLLEGE OF MEDICINE.

The possessors of the Diplomas to be denominated FELLOWS, and to be entitled both in and out of the College to the title of DOCTOR.

The College to be governed by a PRESIDENT and COUNCIL, who are to be elected annually by the FELLOWS in general CONVOCATION.

During the *first year* the Diploma from any University or College of Physicians or Surgeons, shall be deemed a sufficient qualification to entitle the Candidate to the Diploma of this College. But the DIPLOMAS of the LONDON COLLEGE of SURGEONS, dated *subsequently* to Tuesday the 8th of March, the day on which the infamous assault was committed on the MEMBERS, *will not be received.*

The examination of Candidates to take place in PUBLIC, and to be conducted by the PRESIDENT, a COURT of EXAMINERS, and a MEDICAL JURY. Each Candidate to be examined on the *dead body*, in *anatomy*, and *surgery*.

Candidates will not be required to produce any CERTIFICATES whatsoever; as COMPETENCY to undergo a fair and searching practical examination, will be considered the only professional QUALIFICATION sufficient for the attainment of the DIPLOMA.

All Candidates to undergo a general examination in *Anatomy*, *Physiology*, *Pathology*, Surgery, Midwifery, *Practice of Medicine*, Materia Medica, and Chemistry. They will also be required to furnish short translations from the *Greek* and *Latin*, and probably from the *French* and *German* languages.

Whatever may be the determination of Candidates with regard to the line of practice which they may pursue after they have obtained the Diploma,—that is, whether they confine themselves to medicine, surgery, or midwifery,—it will not be admitted by the COURT of EXAMINERS and the JURY that there ought to be any distinction in medical *education*. The FELLOWS will be at liberty to practise in any branch of medical science, but public security will demand that the Candidates display a competent knowledge of the elements of the whole. The sum to be charged for the Diploma will be the lowest that can be named consistent with the maintenance and utility of the College. It is intended that the act of incorporation shall concede to the PRESIDENT, COUNCIL, and FELLOWS of this College, assembled in general convocation, the right to elect the medical officers of the great chartered HOSPITALS; their choice, however, to be subject to the approval of the First Lord of the Treasury; and the authorities of the College to be further empowered to remove such officers in case of incompetence or neglect of duty. A collegiate eleemosynary fund is to be instituted for the support of the widows and orphans of FELLOWS who may be so unfortunate as to leave them unprovided for, and also to render assistance in cases of absolute necessity to any of the FELLOWS themselves, who may be reduced to distress by circumstances over which they may have no control; such claims not to be entertained unless supported by testimonials of high moral character. The eleemosynary collegiate fund to be established and maintained, by life, or annual, contributions from the whole of the FELLOWS; the payments to be regulated by the FELLOWS at the annual collegiate convocation.

---

### XXVIII.

### MEDICAL REFORM.

THERE is, in the medical as well as in the political world, a *mouvement*, as the French term it, and which our medical MAGNATES ought not to hold too cheap. We have advocated rational and liberal reform till we are sick of the theme; but our advocacy was of no avail. It will be seen soon that physical force will be called into action, in aid of arguments. We shall quote a short passage which was written nearly two years ago amid the ruins of Rome, while contemplating the brazen wall or impassable gulph placed between the patrician and plebeian orders of society. It was printed long before the "glorious days" of July, as they are called, but it may apply to medical as well as general politics.

"If the *road* be not left open for industry and talent to acquire property and rank, the lower orders must sink into abject pauperism, or ferment into dangerous rebellion. They have taken the *former* channel in most parts of fair Italy—but with the 'march of intellect,' they will probably run the *latter*, and more fearful course in some other parts of the world. If wealth and power accumulate, beyond all reasonable proportion, in one class, and that the least numerous of society, KNOWLEDGE, which is truly said to be POWER, will ultimately impel the larger and destitute class to organize physical force for the destruction of monopoly and the more equal distribution of wealth. This, it is true will be ROBBERY, attended by bloodshed, and all kinds of crimes. But if Providence permit the hurricane to restore the equilibrium of the atmosphere, while it sweeps whole cities, with all their inhabitants, to destruction, it may sanction the storm of revolution, which subverts the foundations of society, to cure evils that have been growing for ages, fostered by the blind cupidity and the avarice of the human race. The history of the world, and of human nature teaches us that example, or even experience, has little or no influence on man, when his selfish passions are concerned. He will risk all rather than lose a part. When Cato informed Ptolemy, King of Cyprus, that he might retire, with a certain part of his property, he refused. He went out to sea in a ship, with his treasures, determined to sink himself and them, in one common watery grave. His courage amounted to the destruction of himself; but it could not be wound up to the immersion of his riches in the ocean. He sailed back — deposited his money and jewels in safety for his enemies—and then committed suicide! The application of this historical fact to existing circumstances, is not difficult. Our great depositaries of wealth will not concede to measures that may sacrifice a part to preserve the remainder. They will obstinately retain all, like Ptolemy, till the moment when they must lose all!

"Quem Deus vult perdere prius dementat."

"Hitherto the heads of a few have guided the hands of the many—and one channel of thought has fed and set in motion ten thousand springs of action. Ere long, each brain will think for itself, and plan for the common weal. If, in such case, there be any lack of wisdom, it certainly will not be from want of multiplicity of counsellors! Such a state of things is rapidly approaching—nor can it be prevented, even on this oppressed soil, by the Austrian bayonet or Papal crosier. Human wisdom may do much to mitigate the evil, if it be one, by meeting it half way, and lessening the impetus of the revolution. Obstinacy may render the collision of two extremes most awful and destructive."—*Dr. Johnson's Tour through Italy, &c. p.* 182.

